# Effective and rugged analysis of glyphosate, glufosinate, and metabolites in *Tenebrio molitor* larva (mealworms) using liquid chromatography tandem mass spectrometry

**DOI:** 10.1038/s41598-021-96529-8

**Published:** 2021-09-02

**Authors:** Leesun Kim, Sujn Baek, Kyungae Son, Hee-Dong Lee, Dal-Soon Choi, Chang Jo Kim, Hyun Ho Noh

**Affiliations:** Residual Agrochemical Assessment Division, National Institute of Agricultural Sciences, Rural Development Administration, Wanju, 55365 Republic of Korea

**Keywords:** Environmental sciences, Chemistry

## Abstract

*Tenebrio molitor* larva (mealworms) has recently attracted attention as a protein source for food and feed. The larva is generally fed with wheat bran, which can be possibly contaminated with glyphosate. To establish food safe standards, a rugged and effective analytical method for glyphosate, aminomethylphosphonic acid, glufosinate, and their metabolites including 3-methylphosphinico-propionic acid, and *N*-acetyl glufosinate, in mealworms was optimized using liquid chromatography tandem mass spectrometry. An anionic polar pesticide column was used due to its high suitability for glyphosate. Acidified water and acetonitrile were used to extract the target compounds without contribution from various fatty and pigment interferences derived from brownish insects. Seven different clean-up procedures ((1) 50 mg C18 (2) 20 mg C18/Z-sep (3) PRiME hydrophilic-lipophilic balance (HLB) cartridge (4) 75 mg Z-sep, (5) 75 mg Z-sep+, (6) EMR-lipid cartridge, and (7) 50 mg ENVI-Carb) were compared. Due to its simplicity and cost-effectiveness, PRiME HLB was selected for clean-up. The recoveries of the target compounds were ranged from 86 to 96% with < 20% relative standard deviations. Therefore, this simple and effective method can be applied for the two pesticides and their metabolites in other edible insects or high-fat matrices.

## Introduction

Due to their non-selectivity and broad spectrum of activity, glyphosate [*N*-(phosphonomethyl) glycine] and glufosinate [dl-homoalanine-4-yl(methyl) phosphonic acid] have been commonly applied for weed control in agriculture, to control of small shrubs in forestry and urban areas, and harvesting aid as a crop desiccant since their introduction^1^. In Republic of Korea, 28 different types of glyphosate products are applied to the lands of 25 different crops including mandarins to control shrubs or wild grasses. Eleven types of glufosinate are applied to the lands of 81 crops, to remove wild grasses. Neither one is generally used for wheat in Korea, although glyphosate is used as a wheat desiccant in other countries. Therefore, wheat dried with glyphosate desiccant can be possibly imported into Korea for use in animal feed.

Several edible insects including beetles, grasshoppers, honeybees, mealworms, and silkworms are used as alternative protein food sources due to high nutritional values (crude protein 39.3–64.4% and fat 14.4–33.7%) or for medicinal purposes in Republic of Korea^2^. Of these insects, only mealworms are fed with wheat bran, possibly contaminated with glyphosate and glufosinate. There has been concern that the pesticides, glyphosate and glufosinate, can be transferred into mealworms through ingestion of wheat bran. A need existed to set up maximum residue levels (MRLs) of glyphosate and its metabolites because mealworms are already sold in the Korean food markets. However, to the best of our knowledge, no analytical method has not been reported for the analysis of glyphosate, glufosinate, and their metabolites in mealworms. The properties of both amino acid class pesticides and samples with the large amount of fat and protein can make analysts very challenging in analyzing the target compounds in insects^3,4^. In particular, both compounds should be derivatized for gas chromatographic analysis because of their amphotericity, high polarity, non-volatility, and lack of chromophores or fluorophores^5^. However, of various analytical instrumentations, liquid chromatography tandem mass spectrometry (LC–MS/MS) is one of the most common analytical techniques in many laboratories for its accuracy, and low limits of detection (LOD), and no need for derivatization of the target^5–7^.

“Quick Polar Pesticides-Plant Origin (QuPPe-PO) and Animal Origin (QuPPe-AO) methods have been recommended by the EU Reference Laboratories for glyphosate analysis^8^. A previous study modified the QuPPe method to extract glyphosate, glufosinate, bialaphos, and metabolites from human blood^9^. Previous studies optimized the analytical methods of these two pesticides in various food, including baby foods, vegetables^7^, fruits, and drinks^10^ in order to evaluate human dietary exposure. Another study improved the recoveries of 67 pesticides from lipophilic matrices (olive oil, olive, and avocado) by modifying the ratio of solvent/sample to minimize matrix effects originated of various food samples^11^.

After extraction, the clean-up procedure plays an important role in removing interferences that remain in MS/MS analysis. The QuPPe-AO method uses C18 to remove fats from animal- originated samples for analysis of glyphosate^8^. PRiME hydrophilic-lipophilic balance (HLB) and HLB were also mainly used for glyphosate analysis^9,12^. For pesticide analysis, previous studies investigated several adsorbents [C18, Z-sep (zirconium oxide bonded to silica), Z-sep plus (zirconium oxide and C18 dual bonded to silica), and enhanced matrix removal (EMR)-lipid, and PRiME HLB] to effectively remove fat and lipids from highly fatty matrices including avocado^13,14^, cereals^15^, edible insects^3,4^, honey bees^16^, peanut oil^17^, soybean-based products^18^, and vegetable oils^11,19^. However, the pesticides displayed different behaviors in different samples^11^. Magnesium sulfate (MgSO_4_) and primary secondary amine (PSA) are common materials used for adsorbing strong acids or polyacidic compounds mostly derived from aqueous matrices. To effectively eliminate matrix-derived fat and lipids, C18, Z-sep, Z-sep plus, and EMR-lipid were investigated in previous studies^19,20^. A previous study showed that sorbent compositions or ratio in the clean-up process can be more important to improve clean-up efficiency for removal of fatty residues from complex matrices^11^.

This study was designed to optimize a simplified and effective method for the simultaneous analysis of glyphosate, glufosinate, and their metabolites from highly fatty samples using LC–MS/MS. Two columns were also compared for the target analysis. Several extraction solvents and clean-up procedures were tested for more practical and efficient analysis with achieving the reasonable recovery for all the target compounds. The developed method was validated considering limit of quantitation (LOQ), accuracy, and precision.

## Materials and methods

### Chemicals and solvents

Pesticide standards including glyphosate (98.6%), aminomethylphophonic acid (AMPA) (99.9%), gulfosinate (99.2%), 3-methylphosphinico-propionic acid (MPPA) (99.9%), and *N*-acetyl glufosinate (89.4%) were obtained from AccuStandard, Inc. (New Haven, CT, USA). Analytical reagent grade of acetonitrile (MeCN), deionized water (DW), methanol (MeOH), and concentrated formic acid (CH_2_O_2_) were purchased from Merck KGaA (Darmstadt, Hesse, Germany). C18 and ENVI-Carb powder were obtained from Supelco (Bellefonte, PA, USA). OASIS PRiME HLB cartridge (1 mL, 30 mg) was from Waters (Milford, MA, USA). Dispersive solid phase extraction (d-SPE) containing C18 20 mg/Z-sep, Z-sep, and Z-sep +) were obtained from Supelco (Bellefonte, PA, USA). Captiva EMR-lipid cartridge (1 mL, 40 mg) was obtained from Agilent Technologies (Santa Clara, CA, USA).

### Sample pretreatment

Individual stock solutions of standards were prepared in DW (10% MeCN) at a concentration of 1000 µg/mL. Mixture of five standards (glyphosate, AMPA, glufosinate, MPPA, and *N*-acetyl glufosinate) were prepared at 50, 10, and 1 μg/mL by dissolving each stock standard in 10% MeCN solution. The solutions were maintained at 4 °C in polypropylene tubes. Glassware was not used for the experiment at all because glyphosate interacts with glass based on the Quppe method. The matrix matched standards were prepared by mixing the extracting solvent (15 mL DW/100 μL CH_2_O_2_/5 mL MeCN) or blank matrix extract (after clean-up procedure) and standard solutions (10% MeCN) (50:50, v/v) for the calibration curves.

**Figure 1:**
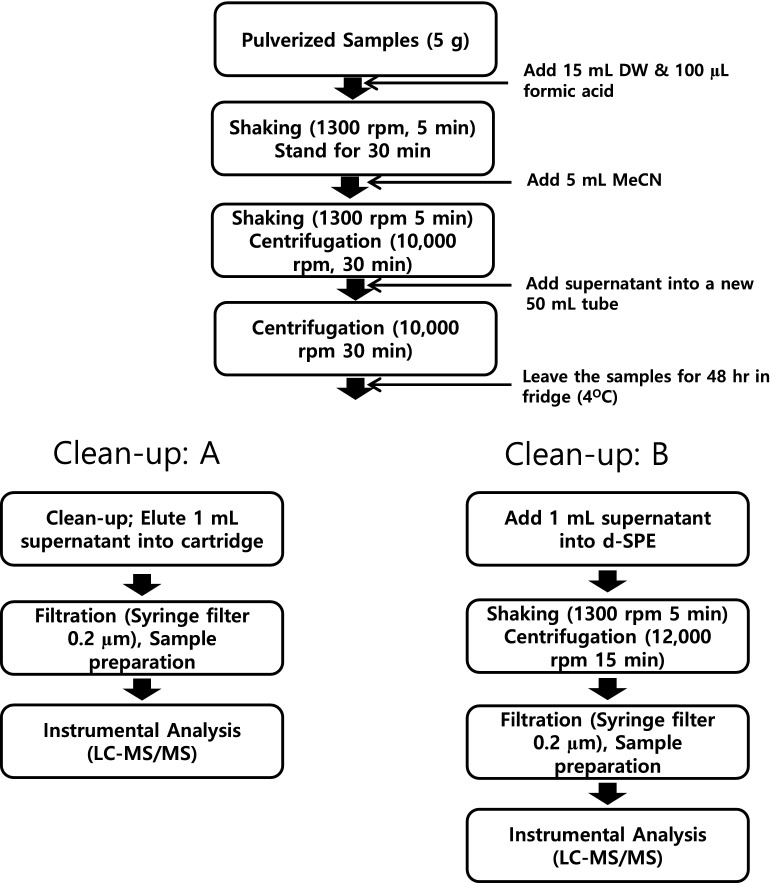
The flow chart for sample preparation for the analysis.

*Tenebrio molitor* larva (mealworms) that are not exposed to either pesticide were kindly provided by Industrial Insect Division, National Institute of Agricultural Sciences (NAS), in Republic of Korea. After the dried samples were pulverized using dry ice and a blender, they were stored in a freezer at − 20°C until the analysis. Figure 1 shows the flow chart for sample preparation procedure. The thawed pulverized sample (5 g) was transferred into a 50 mL of polypropylene centrifuge tube and then moisturized with 15 mL of deionized water acidified with 100 μL of CH_2_O_2_. The samples were allowed to stand for approximately 30 min after being vigorously shaken using a Geno grinder (SPEX. Centiprep 1600 MiniG) for 2 min. Five milliliters of MeCN were added to the sample and the sample was vigorously shaken using a Geno grinder for 2 min. The tube was centrifuged at 10,000 rpm for 30 min at 20°C, and then, the supernatant was transferred into a new tube (50 mL) before another centrifugation (10,000 rpm, 30 min at 20°C). The samples were stored in a fridge (4°C) for 2 days to precipitate lipids and protein residues. The pH of the extract was 5.

For clean-up evaluation, seven different sorbent compositions were tested (Table 1). The supernatant (1 mL) was transferred into a centrifuge tube or d-SPE tube and vigorously shaken before centrifugation (12,000 rpm) at 4 °C for 15 min (Clean-up ﻿A Fig. 1). The supernatant was filtrated through a 0.20 µm membrane filter (MACHEREY–NAGEL, Düren, Germany) before preparation of matrix matched standards and samples for instrumental analysis. For EMR-lipid and PRiME HLB, 1 mL of extract was eluted by SPE cartridge for clean-up (Clean-up B Fig. 1).

**Table 1:** The list of clean-up procedures investigated in this study.

No	Sorbent compositions	Product name
1	50 mg C18	
2	20 mg C18/20 mg Z-sep	
3	Unknown^a^	EMR-lipid
4	30 mg unknown^a^	PRiME HLB
5	75 mg Z-sep	
6	75 mg Z-sep+	
7	50 mg ENVI-Carb	

*PRiME HLB* hydrophilic-lipophilic balance.

^a^Contents are not disclosed by the producer; EMR-lipid: enhanced matrix removal-lipid method.

### Instrumental analysis

The prepared samples were analyzed using LC (AB SCIEX Exion LC) combined with tandem mass spectrometry (AB SCIEX TQ 5500). LC chromatographic separation of the target compounds was performed with anionic polar pesticide columns (APPC, 2.1 mm i.d. × 100 mm L., 5 μm particle size, Waters, MA, USA) with mobile phase A (0.9% CH_2_O_2_ in DW) and mobile phase B (0.9% CH_2_O_2_ in MeCN). The mobile phase gradient program started at 90% at B until 0.5 min, and decreased to 20% B until 1.5 min, changed to 10% B until 4.5 min, stayed at 10% B until 17.5 min, increased to 90% B until 17.6 min and was maintained at 90% B until 23 min. The flow rate was 0.5 mL/min, and the column oven temperature was set at 50°C. The injection volume was 10 μL. The MS/MS was operated in negative electrospray ionization (ESI-) mode and multiple reaction monitoring (MRM) conditions for LC–MS/MS, as listed at Table 2. The MS conditions were as follows: ionspray voltage of 5,500 V, nebulizer gas of 50 psi, curtain gas of 25 psi, drying gas of 50 psi, collision gas of 10 psi, and drying gas temperature of 500°C.

**Table 2:** Multiple reaction monitoring (MRM) conditions of each target compound for LC–MS/MS analysis.

Pesticides	tR (min)	Precursor ion (*m/z*)	Quantitative/confirmation ion *(m/z)*	DP	EP	CE	CXP
Glyphosate	4.4	167.9	62.9/149.7	−45	−10	−32	−15
				−45	−10	−20	−5
AMPA	2.3	110.0	79.0/62.8	−15	−10	−40	−9
				−15	−10	−24	−29
Glufosinate	2.65	179.9	62.9/94.8	−30	−10	−58	−6
				−30	−10	−24	−2
MPPA	3.01	150.9	132.9/62.9	−25	−10	−18	−2
				−25	−10	−42	−6
N-acetyl glufosinate	3.77	222.0	135.9/179.80	−35	−10	−28	−2
				−35	−10	−22	−2

*AMPA* aminomethylphophonic acid; *MPPA* 3-methylphosphinico-propionic acid, *DP* declustering potential, *EP* entrance potential, *CE* collision energy, *CXP* collision cell exit potential.

### Analytical method validation

To evaluate accuracy and precision of the method optimized in this study, mean recovery rate (%) and relative standard deviation (RSD, %), were obtained at two spiking levels of 0.01 and 0.05 mg/kg for LC-analysis. Matrix-matched standards were used to reduce the error caused by instrumental signal enhancement or suppression (matrix effect) by addition of blank matrix extracts to standard solution. The matrix-matched standards were prepared at the levels of 0.5, 1, 2.5, 5, 10, and 25 ng/mL for quantitative analysis. In this study, iso-topically labelled internal standards, which QuPPe method recommended were not used because the recovery results for all the target compounds were consistent without correcting using internal standards. Linearity and limit of quantitation (LOQ) were also assessed by each matrix-matched calibration curve. Matrix-dependent LOQ was determined to be the lowest concentration having an S/N ratio with a quantifier ion peak above 10.

Matrix effect (ME) (%) was calculated using the following equation,

$$\mathrm{ME }\left(\mathrm{\%}\right)=(\frac{slope\,of\,matrix-matched\,standards\,curve}{slope\,of\,solvent\,standards\,curve}-1)\times 100$$

If ME is ~ 0%, there is no matrix effect. If ME is over 0%, an ion suppression occurs and if ME is less than 0%, an ion enhancement occurs. Taken overall, –20% < ME < 20% indicates no influence of matrix effect because 20% ME can be acceptable without correcting for it even though it has an impact on the results.

## Results and discussion

### Optimization of the separation conditions

Glyphosate, glufosinate, and their metabolites are highly water soluble and insoluble in organic solvent and are not retained in conventional reverse phase (a C18 column) chromatography. Previous studies investigated various columns (e.g., hydrophilic interaction chromatography (HILIC column), XBridge amide column (Waters), and anion exchange columns) in glyphosate analysis. A previous study selected HILIC column after comparing HILIC and a amide column for glyphosate analysis in beer, barley tea, and their ingredients because the amide column showed some sample carryover^12^. Another study also used a HILIC column (Oblisc-N) for glyphosate with MS detection in maize, rice, and soybean but showed poor robustness and poor retention time reproducibility^7^. It was also reported that an Acclaim® Mix-mode WAX-1 (reversed-phase/weak anion exchange) was applied to separate glyphosate in water, but after the analysis of 80–100 samples, metal ions accumulated during the analysis caused poor peak shape of glyphosate. Therefore, EDTA solution was used to remove metal ions for long column generation^22^. For glyphosate analysis, an anion exchange (Dionex IonPac AS11) column was also reported to be utilized at a high mobile phase of pH 11 using the alkaline-compatible HPLC components were required (QuPPe method).

**Figure 2:**
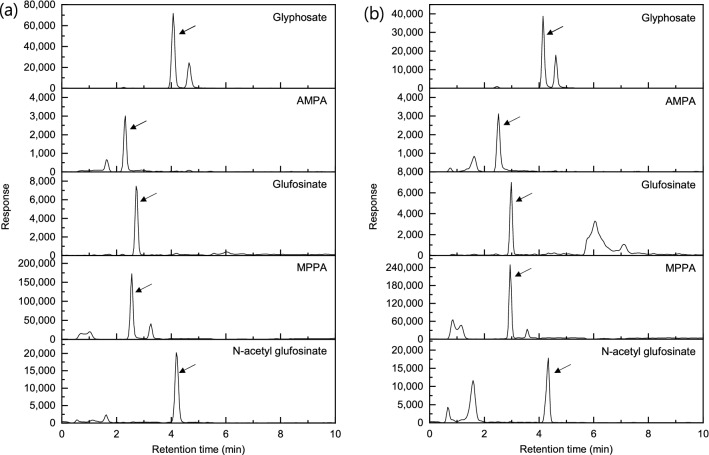
The extracted ion (quantitative ion) chromatograms obtained from one (5 ng/mL) of matrix matched standards of each pesticide (glyphosate (167.9 > 62.9), aminomethylphophonic acid (AMPA) (110.0 > 79.0), glufosinate (179.9 > 62.9), 3-methylphosphinico-propionic acid (MMPA) (150.9 > 132.9), and *N*-acetyl glufosinate (222.0 > 135.9) using **(a)** the anionic polar pesticide column and **(b)** the Torus DEA column.

In this study, two columns (APPC and Torus DEA column (Waters, MA, USA)), specialized in separation of polar compounds such as glyphosate were compared. The extracted ion (quantitative ions described in Table 2) chromatograms obtained from matrix-matched standard (5 ng/mL) of each pesticide are shown in Fig. 2. The matrix-matched standard was prepared with the final extraction and clean-up method optimized in this study. The APPC gave much better response for glyphosate and slightly better response for glufosinate compared with the Torus column. In case of AMPA, peak tailing was observed when the Torus column was used. In addition, considering that the Torus column requires the time-consuming column conditioning (EDTA solution) before and after the analysis, the APPC was selected for this study.

### Optimization of the sample extraction

**Figure 3:**
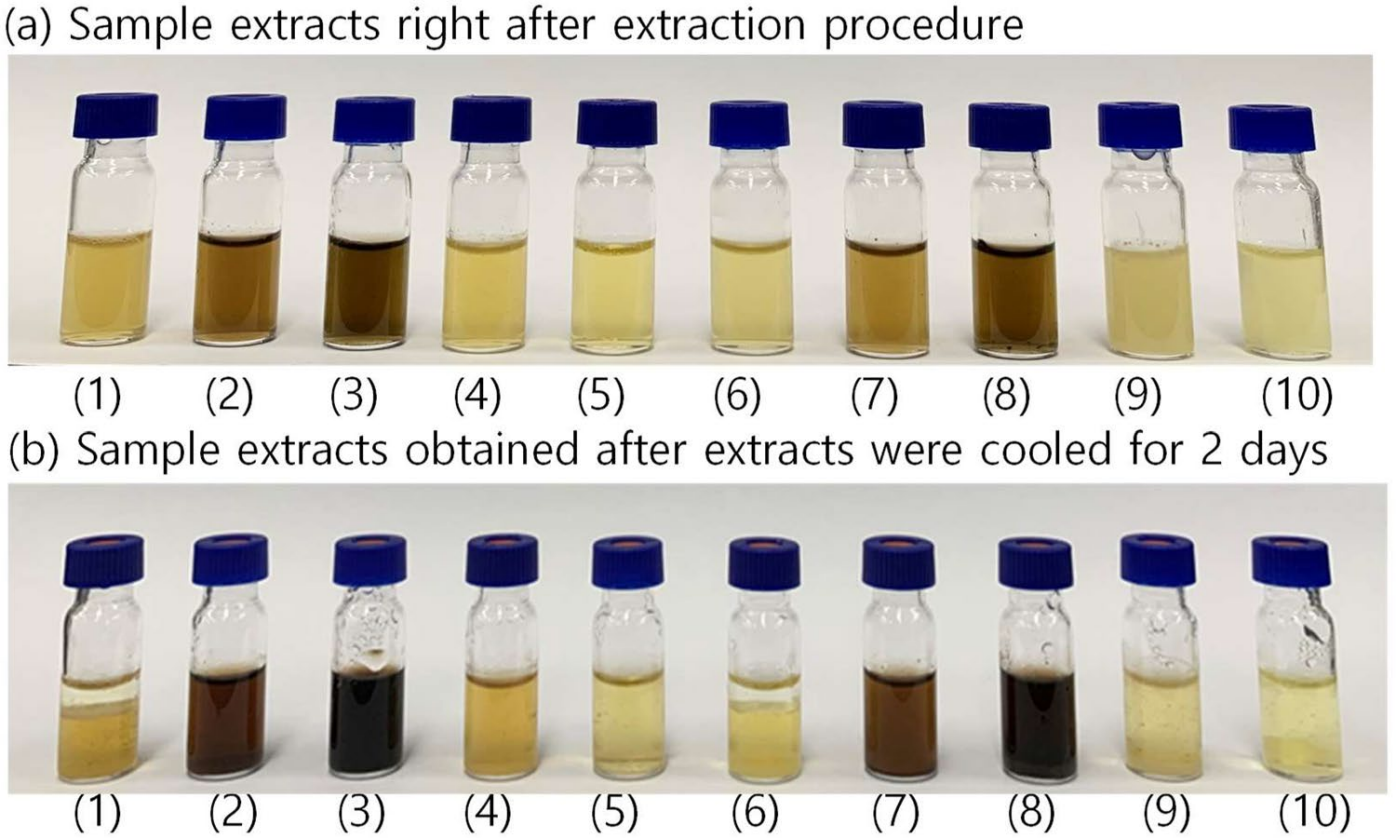
The sample extracts **(a)** right after the extraction was performed and (b) after the samples were stored at 4°C for 48 h. (1) DW (10 mL) and acidified MeCN (10 mL) with 100 µL formic acid (CH_2_O_2_), (2) DW (15 mL) and acidified MeCN (5 mL) with 100 µL CH_2_O_2_, (3) DW (15 mL) and MeCN (5 mL), (4) acidified DW (15 mL) with 100 µL CH_2_O_2_ and MeCN (5 mL), (5) acidified DW (10 mL) with 100 µL CH_2_O_2_ and MeCN (10 mL), (6) DW (10 mL) and acidified MeOH (10 mL) with 100 µL CH_2_O_2_, (7) DW (15 mL) and acidified MeOH (5 mL) with 100 µL CH_2_O_2_, (8) DW (15 mL) and MeOH (5 mL), (9) acidified DW (15 mL) with 100 µL CH_2_O_2_ and MeOH (5 mL), (10) acidified DW (10 mL) with 100 µL CH_2_O_2_ and MeOH (10 mL).

In this study, several extracting solvents were investigated to simultaneously extract glyphosate, AMPA, glufosinate, MMPA, and *N*-acetyl glufosinate. Figure 3 shows the color of extracts (a) right after the samples were extracted with 10 control samples at the spiking level of 0.05 mg/kg were extracted with different ratios of solvents and (b) after the extracts were stored at 4°C for 2 days. Two solvents, MeCN (Fig. 3a 1–5) and MeOH (Fig. 3a 6–10), were compared because MeOH was recommended by the QuPPe-AO method^8^. PRiME HLB cartridges were used for clean-up before the instrumental analysis. Of 10 sample extracts, we did not analyze the extracts 2, 3, 7, and 8 because the insect-derived pigments were not effectively removed by clean-up and could severely contaminate the instrument, in particular, ion source. Our preliminary study did not obtain the recovery data with the dark colored extract. In addition, it was observed that the extracts’ color darkened after they were stored in the fridge for 2 days even though insect-derived fat was precipitated at the bottom. Recoveries of the target compounds listed in the Supplementary Table S1 were obtained from the rest of the extracts at the spiking level of 0.05 mg/kg. Matrix matched standards for each different extract were prepared for quantification for each extract. The results showed that MeCN extracts (68.7–101.5%) gave better recoveries than MeOH ones (16.2–86.7%). Out of three MeCN extracts, extract 4 (acidified DW 15 mL and 5 mL MeCN) gave the best recoveries (92.0 ± 2% for glyphosate, 99.6 ± 4.5% for AMPA, 94.0 ± 10.5% for glufosinate, 83.8 ± 2.9% for MMPA, and 87.0 ± 2.5% for *N*-acetyl glufosinate). Extract 1 (DW 10 mL and MeCN 10 mL) and extract 5 (DW 10 mL and acidified MeCN 10 mL) gave low recoveries (76.7 ± 5.8% and 68.7 ± 16.1%) of AMPA. The results indicate that water content influenced the efficiency of extracting the target compounds, AMPA, in particular. In addition, when water was added to the samples without acid, the water greatly absorbed the insect-derived pigment (brown color) (Fig. 3. sample 3 and 8), indicating that formic acid (100 μL) plays an important role in excluding insect-derived pigment as well as stabilizing the analytes of interest during extraction of target compounds. In extracting pH-sensitive pestcides, formic or acetic acid is often added to the extracting solvent^23,24^.

For pesticide analysis, previous studies applied low-temperature precipitation for fat removal after sample extraction^3,25,26^. Our previous study demonstrated that the recoveries of chlropyrifos-methyl and deltamethrin from mealworms were greatly improved after the sample extracts were stored at –20°C for 24 h^3^. However, in this study, the large amount of fat was effectively removed after the sample extracts were stored at 4°C. Unlike MeCN extract, the predominant portion of the extract was water, and the extract containing fat was frozen at –20°C. Fat was not effectively removed at this low temperature. After 48 h, a large amount of fat was precipitated at the bottom of the vial, as shown in Fig. 3B. Without low temperature precipitation, the recoveries were not even measurable so the data was not presented here.

### Optimization of matrix clean-up

**Figure 4:**
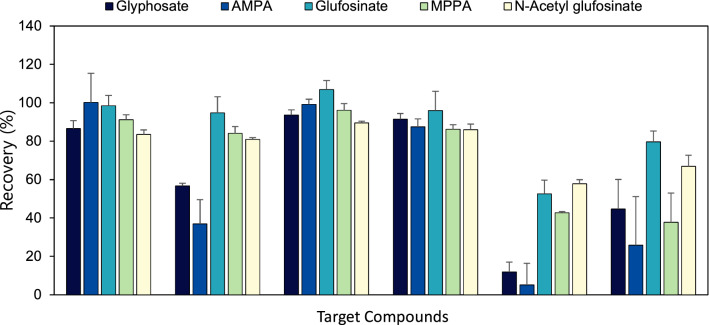
Recoveries of glyphosate, aminomethylphophonic acid (AMPA), glufosinate, 3-methylphosphinico-propionic acid (MMPA), and *N*-acetyl glufosinate obtained from seven different clean-up procedures after extraction of samples with acidified DW (15 mL) with 100 µL formic acid and MeCN (5 mL).

Although previous results for optimizing the extraction method showed satisfactory recoveries for all compounds, additional clean-up procedures were tried for improved ruggedness of the analytical method, as well as to prevent instrumental contamination. Seven different clean-up methods (C18, C18/Z-sep, Z-sep, Z-sep+, EMR-lipid, PRiME HLB, and ENVI-Carb) were compared to optimize the best clean-up method in this study. Before the clean-up procedures, the sample was extracted with acidified DW 15 mL, CH_2_O_2_ 100 μL, and 5 mL MeCN. Figure 4 and Supplementary Table S2 showed that the recoveries of five target compounds were achieved after each clean-up procedure was performed following extraction with acidified DW (15 mL) and MeCN (5 mL). Removal of the fat and protein that remained after acidified water extraction and cooling for 48 h was required because interference can cause signal suppression or enhancement in the MS/MS analysis.

The QuPPe-AO method recommends C18 to remove coextractives from meat samples containing the large amount of fat and proteins for LC–MS/MS analysis ^21^. C18 is one of the most renowned reverse-phase sorbents because it effectively retains fats. A previous study compared C18, PRiME HLB, and no clean-up to remove interference from blood samples. PRiME HLB gave the best recoveries for polar compounds including glyphosate and glufosinate^9^. The composition of PRiME HLB is not revealed by the producer but is recommended for polar analytes including glyphosate. Therefore, C18 and PRiME HLB were investigated in this study as they gave the best recoveries, and the results were not statistically different. One study reported various fat adsorbents for the separation of tryphenylene and chrysene. A mixture of 150 mg MgSO_4_/25 mg PSA/25 mg C18, Z-sep, and EMR-lipid showed similar performance. The C18 mixture was finally chosen because of the higher response of the target compounds and availability of the low-cost and easy-to-use kits ^20^. An EMR-lipid cartridge was also investigated in this experiment because a previous study showed the best recoveries for 255 pesticides^19^. EMR-lipid was a developed sorbent as an alternative of Z-Sep but its composition, as with that of as PRiME HLB, was not also revealed by the producer.

ENVI-Carb is a strong sorbent with a carbon surface consisting of hexagonal ring structures, interconnected and layered into graphitic sheets. Therefore, it has been used to eliminate pigments, polyphenols, and some polar compounds in pesticide analysis ^27,28^. In this study, ENVI-Carb was tested to remove insect-derived pigments. The results showed that the pigments were effectively removed (Supplementary Figure S1). AMPA gave the highest recoveries (91.6 ± 9.3%) but the rest of compounds gave lower recoveries (79.8 ± 9.3% for glyphosate, 84.3 ± 4.5% for glufosinate, 71.9 ± 5.7% for MMPA, and 97.9 ± 8.9% for *N*-acetyl glufosinate), compared to C18, EMR-lipid, and PRiME HLB. The carbon sorbent can retain target compounds except AMPA.

Z-sep and Z-sep+ were investigated in this study, but both absorbed the target compounds. Z-sep in particular retained glyphosate and AMPA much more than Z-sep+ . Recoveries of glyphosate and AMPA obtained after Z-sep clean-up were 11.8 ± 5.1% and 5.1 ± 11.3% respectively. Recoveries of glyphosate and AMPA were obtained after Z-sep+ clean-up were 44.6 ± 15.4% and 25.8 ± 25.4% respectively. In conclusion, C18, EMR-lipid, PRiME HLB showed similar clean-up efficiency. PRiME HLB was selected in this study due to its easy-to-use kits that do not require column conditioning before clean-up.

### Matrix effect (%) and method validation

**Figure 5:**
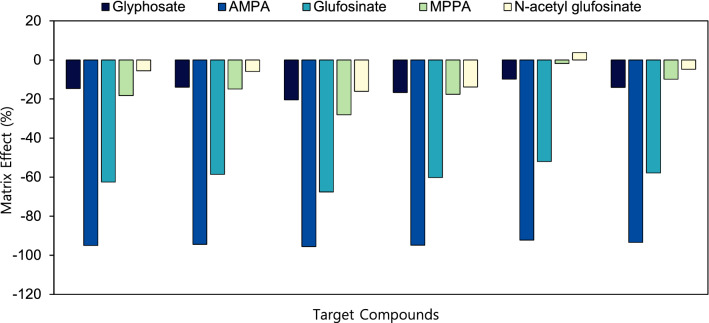
Matrix effects of glyphosate aminomethylphosphonic acid (AMPA), glufosinate, 3-methylphosphinico-propionic acid (MMPA), and *N*-acetyl glufosinate obtained after seven different cleanup procedures were performed after sample extraction with DW (15 mL) acidified with 100 µL formic acid and MeCN (5 mL).

The extraction and clean-up methods optimized in this study was validated. The MEs (%) was determined for five target compounds from mealworms prepared with different types of clean-up methods, as shown in Fig. 5 and Table S2. MEs are of major concern in quantitative LC–MS/MS analysis since they negatively influence accuracy, precision, and sensitivity of an analytical method. To compensate MEs in this study, matrix-matched standards were used for quantification of the target compounds. The clean-up methods showed the similar MEs for each target compound, and each compound displayed a very different ME under fixed conditions. Glyphosate, MPPA, and *N*-acetyl glufosinate gave less than –20% of ME, indicating there were no significant interferences from matrices, and MEs did not cause peak identification or quantification issues. In particular, it was observed that AMPA (–76.0% to –95.5%) and glufosinate (–52.0% to –67.6%) showed strong suppression. In conclusion, these results strongly confirmed the importance of matrix-matched calibration for more accurate quantification of the target compounds. In case of very different matrix sample analysis, standard addition method or iso-topically labelled internal standard is recommended.

The proposed method in this study was assessed for the analysis of target analytes in mealworms. Recovery tests were performed at the two spiking levels of 0.01 mg/kg and 0.05 mg/kg for LC–MS/MS at replicates (n = 3). Calibration curves presented linearity over 1–25 ng/mL with *r*^2^ > 0.99. Retention time for each compound peak was quite consistent though other signals derived from the matrices existed (Fig. 2). Without internal standards, all the analytes gave good recoveries greater than 87% with RSD < 17% for both spiking levels (Table 3). MLOQs for LC–MS/MS were 0.5 μg/L.

**Table 3:** Recoveries of five target compounds at two spiking levels of 0.01 and 0.05 mg/kg.

Target compounds	Spiking level (mg/kg)	Mean (%)	RSD (%)
Glyphosate	0.010	89.8	9.2
	0.050	91.7	1.7
AMPA	0.010	90	16.9
	0.050	105.6	5.4
Glufosinate	0.010	97.9	3.7
	0.050	95.7	3.9
MPPA	0.010	90.5	5.2
	0.050	88.4	1.3
*N*-acetyl glufosinate	0.010	90.7	3.4
	0.050	87.1	1.3

*RSD* relative standard deviation.

## Conclusions

Mealworms have recently been viewed as an up-and-coming protein source for food and feed in Republic of Korea. They are generally fed wheat bran, a byproduct of wheat. In other nations, glyphosate can be used as desiccant when wheat is harvested in other nations, and the herbicide residue in wheat bran can be transferred into mealworms, so the edible insects in the local markets should be monitored for food safety. To that end, this study developed a simple and sensitive analytical method for efficiently determining glyphosate, AMPA, glufosinate, MMPA, and *N*-acetyl-glyfosinate residues in mealworms by LC–MS/MS. A simplified extraction method (acidified water and MeCN) was used to remove the insect-derived pigment, and low- temperature precipitation (4 °C) effectively removed most fats and lipids.

In conclusion, the simplicity and applicability of the method allow routine monitoring of glyphosate, glufosinate, and their metabolites in mealworms and other edible insect matrices, with sufficient selectivity and sensitivity for quantification of low concentrations below the limits established by the Codex Alimentarius. The method also provides the advantages of being effectively, low cost, simplicity, and environmentally friendliness. The sample preparation method does not require many steps and experimental goods, therefore the developed method is cost effective and requires less labor even if it is scaled up to analyze a large amount of market samples. Based on the results, safe management guidelines for glyphosate can be set up for farmers. Future studies can apply this method to develop or optimize the analytical method for glyphosate analysis in the complex matrices with high fat and pigment contents.

## Supplementary Information

Supplementary Information.
