# Supplementary material for: Effective and rugged analysis of glyphosate, glufosinate, and metabolites in Tenebrio molitor larva (mealworms) using liquid chromatography tandem mass spectrometry
Source: Sci Rep. 2021 Sep 2;11:17597. doi: 10.1038/s41598-021-96529-8 (PMC8413439; doi:10.1038/s41598-021-96529-8)
Supplement: Supplementary file 1 — Supplementary Information. [file 41598_2021_96529_MOESM1_ESM.pdf]

Supplementary Information for

Effective and rugged analysis of glyphosate, glufosinate, and their metabolites in *Tenebrio molitor* larva (mealworms) using liquid chromatography tandem mass spectrometry

Leesun Kim[1], Sujn Baek[1], Kyungae Son[1], Hee-Dong Lee[1], Dal-Soon Choi[1], Chang Jo Kim[1], Hyun Hoh[1,*]

[1]*Residual Agrochemical Assessment Division, National Institute of Agricultural Sciences, Rural Development Administration, Wanju, 55365, Republic of Korea*

Contents **Page**

Table S1	Recoveries, regression, and matrix effects of five target compounds obtained using 10 different extraction solvents (at the spiking level of 0.05 mg/kg). PRiME HLB was used for clean-up procedure.	S2,3
Table S2	Recoveries, regressions, and matrix effects of five target compounds obtained using six different cleanup procedures (at the spiking level of 0.05 mg/kg) after extraction samples with acidified DW (15 mL) with 100 µL formic acid and MeCN (5 mL)	S4,5
Figure S1	Sample extracts obtained after (a) PRiME HLB and (b) ENVI-carb (50 mg) with samples extracted with acidified distilled water (15 mL) with 100 µL formic acid and acetonitrile (5 mL)	S6

* Corresponding author. Tel: +82-63-238-2318; fax: +82-63-238-3819.
E-mail address: noh1983@korea.kr (H.H. Noh).

TABLE S1. Recoveries, regression, and matrix effects of five target compounds obtained using 10 different extraction solvents (at the spiking level of 0.05 mg/kg). PRiME HLB was used for clean-up procedure.

No	Extraction solvent	Target Compounds	Regression	ME*(%)	Recoveries (%)				
					1	2	3	Mean	RSD[¥] (%)
1	MeCN	Glyphosate	0.99807	-32.8	92.8	83.3	86.2	87.5	5.6
		AMPA[€]	0.99839	-95.0	81.2	72.3	76.5	76.7	5.8
		Glufosinate	0.99871	-69.7	97.4	93.4	92.5	94.5	2.8
		MPPA[£]	0.99887	-37.0	106.6	96.4	101.5	101.5	5.0
		N-acetyl glufosinate	0.99628	-8.6	91.6	95.2	94.3	93.7	2.0
4		Glyphosate	0.99987	15.0	94.1	91.0	90.8	92.0	2.0
		AMPA	0.99536	-94.3	104.0	95.0	99.7	99.6	4.5
		Glufosinate	0.99854	-55.7	85.8	104.9	91.3	94.0	10.5
		MPPA	0.99944	-12.7	85.0	85.3	81.0	83.8	2.9
		N-acetyl glufosinate	0.99985	-3.1	84.9	86.9	89.2	87.0	2.5
5		Glyphosate	0.99993	-37.7	103.6	76.0	60.0	79.9	27.6
		AMPA	0.99790	-94.8	80.8	66.1	59.2	68.7	16.1
		Glufosinate	0.99913	-65.8	104.9	100.6	71.3	92.3	19.8
		MPPA	0.99926	-36.0	110.8	102.6	74.8	96.0	19.6
		N-acetyl glufosinate	0.99993	-10.7	109.1	94.8	65.6	89.8	24.7
6	MeOH	Glyphosate	0.99991	-21.8	35.3	37.1	38.3	36.9	4.1
		AMPA	0.99416	-96.1	14.9	18.7	15.0	16.2	13.3
		Glufosinate	0.99967	-68.2	87.4	84.3	88.6	86.7	2.6
		MPPA	0.99963	-19.8	86.1	73.9	85.8	81.9	8.5
		N-acetyl glufosinate	0.99961	4.3	84.9	84.4	84.2	84.5	0.4
9		Glyphosate	0.99881	-27.6	80.1	80.2	75.8	78.7	3.2
		AMPA	0.99919	-93.1	84.6	95.6	82.4	87.5	8.1
		Glufosinate	0.99935	-67.6	82.5	81.8	79.6	81.3	1.9

		MPPA	0.99953	-24.8	85.3	83.1	82.3	83.5	1.8
		N-acetyl glufosinate	0.99810	-10.3	80.0	72.0	71.6	74.5	6.4
10		Glyphosate	0.99997	-27.1	73.3	65.9	66.4	68.6	6.0
		AMPA	0.99901	-91.6	78.0	69.1	62.3	69.8	11.3
		Glufosinate	0.99991	-60.5	76.4	71.7	74.1	74.1	3.2
		MPPA	0.99994	-17.0	72.9	67.1	73.7	71.3	5.0
		N-acetyl glufosinate	0.99986	-1.8	76.6	64.1	67.1	69.3	9.4

1. DW (10 mL) and Acidified MeCN (10 mL) with 100 µL formic acid
4. Acidified DW (15 mL) with 100 µL formic acid and MeCN (5 mL)
5. Acidified DW (10 mL) with 100 µL formic acid and MeCN (10 mL)
6. DW (10 mL) and Acidified MeOH (10 mL) with 100 µL formic acid
9. Acidified DW (15 mL) with 100 µL formic acid and MeOH (5 mL)
10. Acidified DW (10 mL) with 100 µL formic acid and MeOH (10 mL)

*ME: matrix effect; ¥RSD: relative standard deviation; €AMPA: aminomethylphophonic acid; £MMPA: 3-methylphosphinico-propionic acid

TABLE S2. Recoveries, regressions, and matrix effects of five target compounds obtained using six different cleanup procedures (at the spiking level of 0.05 mg/kg) after extraction samples with acidified distilled water (15 mL) with 100 µL formic acid and acetonitrile (5 mL)

Cleanup Procedure	Target Compounds	Regression	ME*(%)	Recoveries (%)				
				1	2	3	mean	RSD¥ (%)
50 mg C18	Glyphosate	0.99998	-14.6	82.8	87.1	89.8	86.6	4.1
	AMPA	0.99956	-95.0	96.3	87.3	117.0	100.2	15.2
	Glufosinate	0.99989	-62.5	96.0	94.9	104.6	98.5	5.4
	MPPA	0.99982	-18.3	91.9	88.5	93.0	91.1	2.6
	N-acetyl glufosinate	0.99995	-5.6	84.2	81.4	85.1	83.5	2.3
20 mg C18 /20 mg Z-sep	Glyphosate	0.99984	-13.9	56.9	56.0	57.4	56.7	1.3
	AMPA	0.99966	-94.5	32.0	37.8	41.1	37.0	12.5
	Glufosinate	0.99942	-58.6	86.2	96.3	101.8	94.8	8.3
	MPPA	0.99997	-14.9	80.9	86.9	84.4	84.1	3.6
	N-acetyl glufosinate	0.99996	-5.9	81.7	80.7	80.4	80.9	0.9
EMR-lipid	Glyphosate	0.99986	-20.4	92.0	92.2	96.5	93.6	2.7
	AMPA	0.99925	-95.5	96.5	101.9	99.1	99.2	2.7
	Glufosinate	0.99922	-67.6	101.2	108.8	110.7	106.9	4.7
	MPPA	0.99989	-28.1	92.3	98.2	97.8	96.1	3.4
	N-acetyl glufosinate	0.99982	-16.1	89.2	89.2	90.4	89.6	0.8
PRiME HLB	Glyphosate	0.99986	-16.7	94.0	91.7	88.6	91.4	2.9
	AMPA	0.99482	-94.8	88.6	83.4	90.4	87.4	4.2
	Glufosinate	0.99869	-60.2	87.9	106.7	93.3	96.0	10.1
	MPPA	0.99953	-17.6	87.8	87.0	84.0	86.3	2.3
	N-acetyl glufosinate	0.99992	-13.8	83.7	85.5	88.7	86.0	2.9
75 mg Z-sep	Glyphosate	0.99982	-9.8	11.2	12.3	12.0	11.8	5.1
	AMPA	0.99829	-92.2	4.4	5.5	5.3	5.1	11.3

	Glufosinate	0.99841	-52.0	54.3	48.4	55.2	52.6	7.1
	MPPA	0.99989	-1.8	42.8	42.3	42.9	42.7	0.8
	N-acetyl glufosinate	0.99974	3.8	56.6	57.8	59.0	57.8	2.1
75 mg Z-sep+	Glyphosate	0.99980	-14.0	43.6	52.0	38.3	44.6	15.4
	AMPA	0.99810	-93.4	26.1	32.1	19.1	25.8	25.4
	Glufosinate	0.99895	-57.9	74.6	83.4	80.9	79.7	5.7
	MPPA	0.99996	-9.8	39.0	42.8	31.5	37.8	15.2
	N-acetyl glufosinate	0.99996	-4.8	65.9	71.1	63.6	66.9	5.8
50 mg Envi-Carb	Glyphosate	0.99689	-16.6	86.53	80.82	72.02	79.8	9.2
	AMPA	0.99798	-76.0	97.44	104.4	86.58	96.1	9.3
	Glufosinate	0.99998	-57.4	87.63	85.04	80.19	84.3	4.5
	MPPA	0.99998	-0.2	74.51	73.95	67.1	71.9	5.7
	N-acetyl glufosinate	0.99998	10.1	82.44	76.33	68.97	75.9	8.9

*ME: matrix effect; ¥RSD: relative standard deviation; €AMPA: aminomethylphophonic acid; £MMPA: 3-methylphosphinico-propionic acid

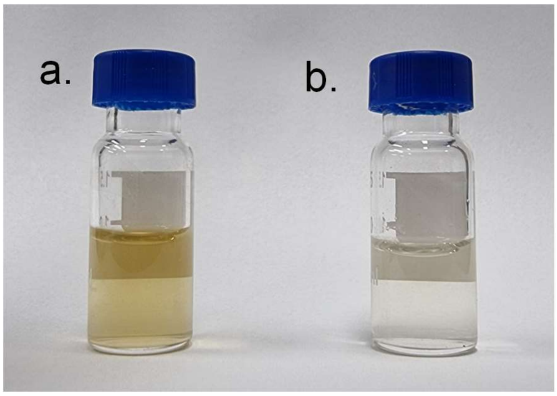

Figure S1. Sample extracts obtained after (a) PRiME HLB and (b) ENVI-carb (50 mg) with samples extracted with acidified distilled water (15 mL) with 100 µL formic acid and acetonitrile (5 mL)
